# Postpartum Breast Cancer and Survival in Women With Germline *BRCA* Pathogenic Variants

**DOI:** 10.1001/jamanetworkopen.2024.7421

**Published:** 2024-04-19

**Authors:** Zhenzhen Zhang, Shangyuan Ye, Sarah M. Bernhardt, Heidi D. Nelson, Ellen M. Velie, Virginia F. Borges, Emma R. Woodward, D. Gareth R. Evans, Pepper J. Schedin

**Affiliations:** 1 Division of Oncological Sciences, Oregon Health & Science University, Portland; 2 Knight Cancer Institute, Oregon Health & Science University, Portland; 3 Biostatistics Shared Resource, Knight Cancer Institute, Oregon Health & Science University, Portland; 4 Department of Cell, Developmental & Cancer Biology, Oregon Health & Science University, Portland; 5 Kaiser Permanente Bernard D. Tyson School of Medicine, Pasadena, California; 6 Zilber College of Public Health, University of Wisconsin-Milwaukee, Milwaukee; 7 Departments of Medicine and Pathology, Medical College of Wisconsin, Milwaukee; 8 Young Women’s Breast Cancer Translational Program, Division of Medical Oncology, Department of Medicine, University of Colorado Anschutz Medical Campus, Aurora; 9 Manchester Centre for Genomic Medicine, Manchester Academic Health Sciences Centre, Division of Evolution Infection and Genomic Science, St Mary’s Hospital, University of Manchester, Manchester, United Kingdom; 10 Prevent Breast Cancer Centre, University Hospital of South Manchester NHS Trust, Wythenshawe, Manchester, United Kingdom; 11 Manchester Centre for Genomic Medicine, St Mary’s Hospital, Central Manchester University Hospitals NHS Foundation Trust, Manchester, United Kingdom; 12 Manchester Breast Centre, University of Manchester, Manchester, United Kingdom

## Abstract

**Importance:**

In young-onset breast cancer (YOBC), a diagnosis within 5 to 10 years of childbirth is associated with increased mortality. Women with germline *BRCA1/2* pathogenic variants (PVs) are more likely to be diagnosed with BC at younger ages, but the impact of childbirth on mortality is unknown.

**Objective:**

To determine whether time between most recent childbirth and BC diagnosis is associated with mortality among patients with YOBC and germline *BRCA1/2* PVs.

**Design, Setting, and Participants:**

This prospective cohort study included women with germline *BRCA1/2* PVs diagnosed with stage I to III BC at age 45 years or younger between 1950 and 2021 in the United Kingdom, who were followed up until November 2021. Data were analyzed from December 3, 2021, to November 29, 2023.

**Exposure:**

Time between most recent childbirth and subsequent BC diagnosis, with recent childbirth defined as 0 to less than 10 years, further delineated to 0 to less than 5 years and 5 to less than 10 years.

**Main Outcomes and Measures:**

The primary outcome was all-cause mortality, censored at 20 years after YOBC diagnosis. Mortality of nulliparous women was compared with the recent post partum groups and the 10 or more years post partum group. Cox proportional hazards regression analyses were adjusted for age, tumor stage, and further stratified by tumor estrogen receptor (ER) and *BRCA* gene status.

**Results:**

Among 903 women with *BRCA* PVs (mean [SD] age at diagnosis, 34.7 [6.1] years; mean [SD] follow-up, 10.8 [9.8] years), 419 received a BC diagnosis 0 to less than 10 years after childbirth, including 228 women diagnosed less than 5 years after childbirth and 191 women diagnosed 5 to less than 10 years after childbirth. Increased all-cause mortality was observed in women diagnosed within 5 to less than 10 years post partum (hazard ratio [HR], 1.56 [95% CI, 1.05-2.30]) compared with nulliparous women and women diagnosed 10 or more years after childbirth, suggesting a transient duration of postpartum risk. Risk of mortality was greater for women with ER-positive BC in the less than 5 years post partum group (HR, 2.35 [95% CI, 1.02-5.42]) and ER-negative BC in the 5 to less than 10 years post partum group (HR, 3.12 [95% CI, 1.22-7.97]) compared with the nulliparous group. Delineated by *BRCA1* or *BRCA2*, mortality in the 5 to less than 10 years post partum group was significantly increased, but only for *BRCA1* carriers (HR, 2.03 [95% CI, 1.15-3.58]).

**Conclusions and Relevance:**

These findings suggest that YOBC with germline *BRCA* PVs was associated with increased risk for all-cause mortality if diagnosed within 10 years after last childbirth, with risk highest for ER-positive BC diagnosed less than 5 years post partum, and for ER-negative BC diagnosed 5 to less than 10 years post partum. *BRCA1* carriers were at highest risk for poor prognosis when diagnosed at 5 to less than 10 years post partum. No such associations were observed for *BRCA2* carriers. These results should inform genetic counseling, prevention, and treatment strategies for *BRCA* PV carriers.

## Key Points

**Question:**

Is a postpartum diagnosis an independent risk factor associated with mortality among patients with young-onset breast cancer with germline *BRCA1/2* pathogenic variants (PVs)?

**Findings:**

This cohort study including 903 women with *BRCA* germline PVs found that a breast cancer diagnosis less than 10 years post partum was associated with higher risk of mortality compared with nulliparous women and women diagnosed at least 10 years post partum. Increased risk after childbirth varied, with highest risk at less than 5 years for women with ER-positive breast cancer vs 5 to less than 10 years for women with ER-negative breast cancer, and *BRCA1* carriers had peak risk of mortality 5 to less than 10 years post partum, with no associations observed for *BRCA2* carriers.

**Meaning:**

These findings suggest that a breast cancer diagnosis within 10 years of childbirth was independently associated with increased risk for mortality in patients with germline *BRCA1/2* PVs, especially for carriers of *BRCA1* PVs.

## Teaser

This cohort study assesses whether time between most recent childbirth and breast cancer diagnosis is associated with mortality among patients with young-onset breast cancer and germline *BRCA1/2* pathogenic variants.

## Introduction

In the United Kingdom^1^ and the United States,^2^ breast cancer (BC) diagnosed at age 45 years and younger (young-onset BC [YOBC]) accounts for approximately 10% of all newly diagnosed invasive BC. The incidence of YOBC is even higher in other countries, accounting for approximately 19% of all newly diagnosed invasive BC worldwide.^1^ Furthermore, the incidence trend of YOBC has been gradually increasing worldwide for decades.^3,4,5,6^ This increasing incidence is likely unrelated to increased mammographic detection, as most patients diagnosed with YOBC are too young for routine screening.^7,8^ Rather, increased incidence may be due, at least in part, to changes in reproductive factors, including pregnancies occurring at older ages.^9^ Although overall treatment has improved outcomes for patients with BC at all ages,^10^ those with YOBC continue to experience elevated mortality rates and have had only modest improvements in treatment efficacy.^11^ Importantly, compared with later age–onset BC, YOBC is enriched with poor prognostic tumor features^12,13,14,15,16^ and is associated with higher mortality^6,11,15,17,18^

An emerging body of work suggest that the postpartum period is a high-risk window for initiation of new cancers and the rapid progression of subclinical lesions to cancers with metastatic phenotypes.^19,20,21,22,23^ Meta-analyses of YOBC showed a postpartum diagnosis up to 10 years after childbirth was consistently associated with increased risk of distant metastasis and death.^19,20,24^ These BCs are defined as postpartum BC (PPBC).^25^ Given that proximity to recent childbirth is such a strong factor associated with BC metastasis and survival in the general population,^19,20,22,23,24,26,27,28,29,30^ the question of whether women with hereditary pathogenic variants (PVs) in BC predisposing genes have similarly poorer prognosis merits investigation. Of approximately 2.3 million women worldwide diagnosed with BC each year,^31^ 5% to 6% have hereditary gene PVs, with *BRCA* PVs being dominant, accounting for approximately half of BC with hereditary PVs.^32,33^ PVs in *BRCA1* and *BRCA2* genes were discovered in 1994^34^ and 1995,^35^ respectively, and both genes encode tumor suppression proteins directly linked to homologous recombination repair of DNA.^36^ The risk of developing BC is approximately 72% for *BRCA1* and 69% for *BRCA2* PV carriers,^37^ while in the general population, the lifetime risk of developing BC is 13%. The peak incidence for *BRCA* carriers is also younger than the general population, occurring in women aged 41 to 50 years for *BRCA1* carriers and 51 to 60 years for *BRCA2* carriers.^37^

To better understand the association of recent childbirth with prognosis of *BRCA1/2* YOBC, we assessed whether time between recent childbirth and BC diagnosis was associated with increased mortality in patients with *BRCA1/2* BC enrolled in the Manchester UK Centre for Genomic Medicine and Family History Clinic.^38^ Evaluating potential associations between recent childbirth and survival outcomes could lead to improved strategies to prevent and treat YOBC in carriers of germline *BRCA* PVs.

## Methods

The parent study was approved by the University of Manchester ethics review board. All women consented to the parent study. We received deidentified data, and the Oregon Health & Science University institutional review board approved this cohort study as exempt for the use of secondary data analyses. This study followed the Strengthening the Reporting of Observational Studies in Epidemiology (STROBE) reporting guideline.

### Database Setting

The study population is part of a prospectively maintained database of carriers of *BRCA* PVs at the Manchester Centre for Genomic Medicine, United Kingdom.^38,39^ Women with a family or personal history of breast or ovarian cancer were referred to the Family History Clinic and the Manchester Centre for Genomic Medicine, both founded in 1987.^38^ Parity data were collected through questionnaires, and detailed pedigrees were administered during clinic visits. Testing for *BRCA1/2* PVs began in 1996. Women identified with germline *BRCA1* or *BRCA2* pathogenic variants (confirmed using American College of Medical Genetics and Genomics/Association for Molecular Pathology criteria) are the source of the study population. Individuals heterozygous for PVs and their first-degree relatives were entered into a dedicated database.^39^ Many additional heterozygous and obligate carriers were identified by cascading.^40^ Follow-up information was collected through medical record review and from the National Cancer Registration and Analysis Service. BC subtype information was obtained through abstraction of patient pathology reports.

### Participants

As of November 2021, a total of 1712 unrelated families with germline *BRCA1/2* PVs and 3588 women heterozygous for *BRCA1/2* PVs were identified in the database. After excluding 1654 individuals without cancer and 6 individuals with stage IV cancer or missing data on BC status, a total of 1928 patients with BC and *BRCA1/2* PVs were identified (eFigure 1 in Supplement 1). Prophylactic mastectomy and oophorectomy surgery prior to BC diagnosis has been shown to reduce breast cancer mortality among women with heterozygous *BRCA1/2*; however our data did not suggest a consistent survival benefit among these individuals (eFigure 2 in Supplement 1). Nonetheless, we excluded 65 patients who had oophorectomy or mastectomy before BC diagnosis. Additionally, we excluded 50 patients with ductal carcinoma in situ; 680 patients diagnosed at age older than 45 years; 183 patients without diagnosis date, date of first childbirth, or date of most recent childbirth; 40 patients diagnosed during pregnancy; and 7 patients diagnosed before 1950. This resulted in a final analytical cohort of 903 eligible patients with nonmetastatic (stage I-III) BC with germline *BRCA1* and *BRCA2* PVs, with complete data on time since recent childbirth, and who were diagnosed at age at least 15 and no older than 45 years between 1950 and 2021 (eFigure 1 in Supplement 1). The mean (SD) follow-up time was 10.8 (9.8) years (IQR, 2.8-16.1 years).

Within this cohort, 224 women were nulliparous at the time of their BC diagnosis and 10 of these women had a first birth after their BC diagnosis. We conducted a sensitivity analysis comparing the survival difference between these 10 women and the rest of the nulliparous women and found no statistically significant differences (eFigure 3 in Supplement 1). Thus, we included these 10 women with subsequent childbirth in the nulliparous group. Women with BC were followed-up until November 4, 2021, or until death, whichever came first.

### Outcomes, Exposures, and Covariates

The primary outcome for this study was all-cause mortality. Survival duration was calculated as the time between the date of initial BC diagnosis and the date of death or the date of last contact, up to the study cutoff date (November 4, 2021). Follow-up time was censored at 20 years.

The main exposure was the time interval between most recent childbirth and BC diagnosis, using previous definitions of PPBC, defined as diagnosis less than 10 years after most recent childbirth.^25^ Analyses were performed on the 3 groups (nulliparous, PPBC 0 to <10 years, and ≥10 years since recent childbirth). Also, where sample sizes permitted, we further delineated the PPBC group into 0 to less than 5 years and 5 to less than 10 years subgroups, as some studies have reported that closer proximity to recent childbirth was associated with worse prognosis.^22,23^

Covariates considered included tumor estrogen receptor (ER) status (ER-positive or ER-negative), clinical stage at BC diagnosis, patient age at diagnosis, year of BC diagnosis, parity, age at first full-term birth, age at last full-term birth, *BRCA1* vs *BRCA2* PV status, and type of *BRCA* PV (ie, copy number variants, truncating, splice site, missense PVs, and promoter alterations). *BRCA1/2* PVs were assessed with all exons sequencing before 2014 and by next-generation sequencing with multiple ligation dependent probe amplification for whole exon deletions or duplications after 2014.

### Statistical Analysis

We used χ^2^ tests for each categorical variable (ER status, tumor size, histology grade, stage, year of diagnosis, parity status, age at first full-term birth at time of diagnosis, age at last full-term birth at time of diagnosis, age at menarche, *BRCA1/2* PVs, type of PVs) and the Kruskal-Wallis test for continuous variables (age at diagnosis). The Kaplan-Meier method was used to calculate survival estimates, and the log-rank test was used to compare survival curves by time since recent childbirth group. Multivariate Cox proportional hazards regression was applied to identify factors associated with the overall mortality. The proportionality assumption was tested using Schoenfeld residuals, with *BRCA* or ER status showing a nonconstant hazard over time. To account for this, the data were stratified by *BRCA* PV type or ER status where appropriate. The models are based on univariate analyses of the time since recent childbirth groups followed by multivariate models that include the following covariates: age at BC diagnosis, tumor stage at diagnosis, BC ER status, and *BRCA* PV type. Diagnosis year was not included in the main adjustment because there were no significant differences in diagnosis year in association with mortality in this dataset (eTable 1 in Supplement 1). Furthermore, there were no significant differences in diagnosis year with mortality among nulliparous women (eFigure 4 in Supplement 1). Cox proportional hazards were used to calculate mortality hazard ratios (HRs) and 95% CIs.

By definition, data for the main exposure variable, time since recent childbirth, were available for all patients in the analytic cohort. However, several covariates included missing values (eTable 2 in Supplement 1). The distribution of data for time since recent childbirth (main exposure) and mortality (outcome), were compared between patients with and without missing values for the covariates (eTable 3 in Supplement 1). There were no significant differences in the number of individuals with and without missing data when comparing between the time since recent childbirth groups. However, patients without missing values had significantly better rates of survival than patients with missing values, an observation consistent with findings from large national cancer registries.^41^ Since exclusion of patients with missing variables from analyses may introduce unintended bias and underestimate BC mortality,^41^ patients with missing data for each covariate were considered as a distinct category.

The analyses of potential modifiers of the association between time since recent childbirth and survival included parity (nulliparous, 1, ≥2), age of first full-term birth (nulliparous, <25 years, ≥25 years), and age at last full-term birth (nulliparous, <25 years, ≥25 years). All statistical analyses were conducted using R software version 4.1.1 (R Project for Statistical Computing) or SAS software version 9.4 (SAS Institute). Tests of statistical significance were determined using 2-tailed tests, and *P* = .05 was considered statistically significant. Data were analyzed from December 3, 2021, to November 29, 2023.

## Results

### Patient Characteristics

The analytic cohort included 903 women with stage I-III BC diagnosed at age 45 years or younger (mean [SD] age, 37.3 [5.4] years) (eFigure 1 in Supplement 1). The mean (SD) follow-up time was 10.8 (9.8) years (IQR, 2.8-16.1 years). Participant demographic and clinical characteristics are summarized in Table 1. There were statistically significant differences in age at diagnosis, tumor histology grade, year of diagnosis, age at first full-term birth, and age at last full-term birth across the time since recent childbirth groups (Table 1). However, there were no differences in ER status, tumor size, stage, parity (among parous groups), age at menarche, distribution of *BRCA1 *or *BRCA2* PVs, or type of PV (ie, copy number variants, truncating, splice site, missense PVs, and promoter alterations) across groups (Table 1). Further demographic and clinical characteristics of the study population comparing *BRCA1* vs *BRCA2* groups are presented in eTable 4 in Supplement 1. Among women with ER-positive BC, *BRCA2* was the dominant *BRCA* PV type, ranging from 62.3% among the nulliparous group to 75.0% among the PPBC at less than 5 years group; the frequency distributions of *BRCA2* among PPBC 5 to less than 10 years and parous BC at 10 years or more groups were both 70.3% (*P* = .56). Among women with ER-negative BC, *BRCA1* PVs were dominant, ranging from 74.6% in the PPBC at 5 to less than 10 years group to 87.3% in the PPBC at less than 5 years group; the frequency distribution of *BRCA1* among the nulliparous group was 81.1% and among the parous BC at 10 years or more group was 80% (*P* = .08). These results suggest significant associations between *BRCA1/2* PVs and ER tumor subtype, consistent with previous reports.^42^ We did not observe differences in ER tumor subtypes among the time since recent childbirth groups in *BRCA* carriers. Furthermore, the proportions of women with *BRCA1* vs *BRCA2* PVs did not differ among the time since recent childbirth groups.

**Table 1.** Demographic and Clinical Characteristics of the Analytic Cohort by Time Since Recent Childbirth

Characteristic	Patients, No. (%)				*P* value
	Nulliparous (n = 224)^a^	PPBC <5 y (n = 228)	PPBC 5 to <10 y (n = 191)	Parous ≥10 y (n = 260)	
Age at diagnosis, mean (SD), y	34.7 (6.1)	35.2 (4.3)	37.5 (4.4)	41.3 (3.3)	<.001^b^
Estrogen receptor status					
Positive	61 (45.2)	64 (47.4)	42 (41.6)	64 (46.0)	.84^c^^,^^d^
Negative	74 (54.8)	71 (52.6)	59 (58.4)	75 (54.0)	
Missing	89	93	90	121	
Tumor size, cm					
0.1 to ≤2.0	51 (55.4)	46 (51.7)	47 (69.1)	49 (52.1)	.39^c^^,^^d^
>2.0 to ≤5.0	40 (43.5)	42 (47.2)	20 (29.4)	44 (46.8)	
>5.0	1 (1.1)	1 (1.1)	1 (1.5)	1 (1.1)	
Missing	132	139	123	166	
Histology grade					
I	0	2 (1.3)	2 (1.8)	2 (1.3)	<.001^c^^,^^d^
II	21 (14.8)	19 (12.6)	31 (27.2)	32 (20.8)	
III	128 (85.2)	130 (86.1)	81 (71.1)	121 (77.9)	
Missing	75	77	77	105	
Stage					
1	42 (46.2)	32 (35.6)	37 (52.1)	39 (38.6)	.23^c^^,^^d^
2	47 (51.6)	53 (58.9)	29 (40.8)	57 (56.4)	
3	2 (2.2)	5 (5.6)	5 (7.0)	5 (5.0)	
Missing	133	138	120	159	
Year of diagnosis					
<1980	10 (4.5)	32 (14.0)	33 (17.3)	32 (12.3)	<.001^c^
1980-1990	14 (6.3)	33 (14.5)	23 (12.0)	50 (19.2)	
1990-2000	58 (25.9)	63 (27.6)	53 (27.8)	72 (27.7)	
2000-2010	63 (28.1)	47 (20.6)	43 (22.5)	67 (25.8)	
>2010	79 (35.3)	53 (23.3)	39 (20.4)	39 (15.0)	
Parity status at diagnosis					
1	NA	47 (20.6)	36 (18.8)	54 (20.8)	.87^c^
2	NA	106 (46.5)	92 (48.2)	130 (50.0)	
≥3	NA	75 (32.9)	63 (33.0)	76 (29.2)	
Age at first full-term birth at time of diagnosis, y					
<21	NA	29 (12.7)	36 (18.85)	86 (33.1)	<.001^c^
21-29	NA	112 (49.1)	119 (62.3)	162 (62.3)	
30-39	NA	85 (37.3)	36 (18.85)	12 (4.6)	
≥40	NA	2 (0.9)	0	0	
Age at last full-term birth at time of diagnosis, y					
<21	NA	0	3 (1.6)	22 (8.5)	<.001^c^
21-29	NA	53 (23.2)	79 (41.4)	177 (68.1)	
30-39	NA	162 (71.1)	108 (56.5)	60 (23.1)	
≥40	NA	13 (5.7)	1 (0.5)	1 (0.4)	
Age at menarche, y					
≤13	61 (67.0)	63 (64.9)	47 (62.7)	77 (64.2)	.95^c^^,^^d^
>13	30 (33.0)	34 (35.1)	28 (37.3)	43 (35.8)	
Missing	133	131	116	140	
PVs					
* BRCA1*	122 (54.5)	137 (60.1)	105 (55.0)	145 (55.8)	.62^c^
* BRCA2*	102 (45.5)	91 (39.9)	86 (45.0)	115 (44.2)	
Type of PV					
Copy number variants (large deletion + large rearrangement)	31 (13.8)	35 (15.4)	20 (10.5)	26 (10.0)	.14^c^
Truncating	167 (74.6)	169 (74.1)	161 (84.3)	208 (80.0)	
Splice site	16 (7.1)	15 (6.6)	5 (2.6)	16 (6.2)	
Missense	9 (4.0)	5 (2.2)	5 (2.6)	9 (3.5)	
Promotor	1 (0.4)	4 (1.8)	0	1 (0.4)	

Abbreviations: NA, not applicable; PPBC, postpartum breast cancer; PV, pathogenic variant.

^a^ Ten Patients who have birth after diagnosis were included in the nulliparous group.

^b^ Kruskal-Wallis test.

^c^ χ^2^ test or Fisher Exact test.

^d^ Missing value categories were excluded from *P* value calculation.

### Associations of Time Since Recent Childbirth and All-Cause Mortality

**Figure 1.**
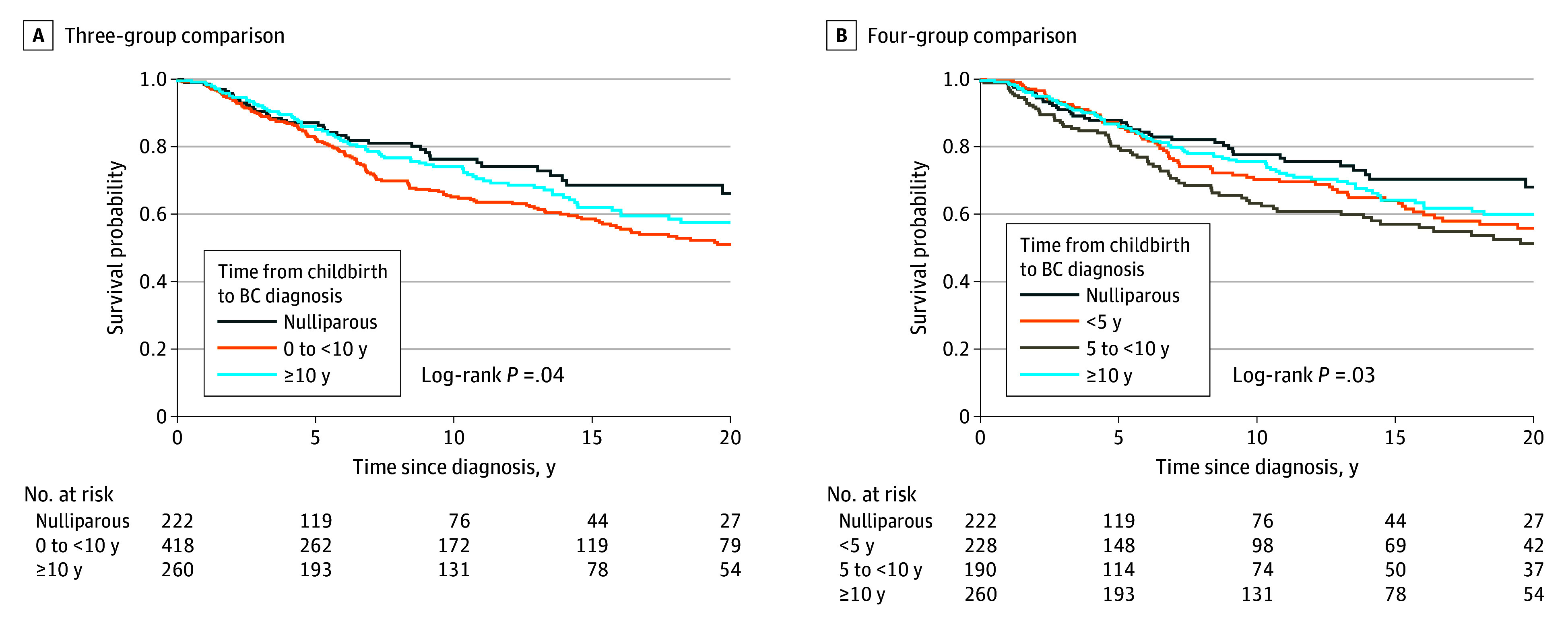
Survival Outcomes by Time Since Recent Childbirth for all *BRCA1* and *BRCA2* Germline Pathogenic Variant Carriers With Breast Cancer (BC)

We next evaluated the association of time since recent childbirth with overall mortality among women with germline *BRCA* BC using the Kaplan-Meier method. We found that a PPBC diagnosis at less than 10 years (Figure 1A), especially at 5 to less than 10 years (Figure 1B), was associated with increased risk of overall mortality compared with nulliparous women. The group with BC diagnosis at least 10 years after childbirth had no significant difference compared with the nulliparous group (Figure 1).

To determine the magnitude of the increased risk of overall mortality associated with PPBC, we conducted univariate Cox proportional hazards regression analyses. When comparing PPBC at 5 to less than 10 years vs nulliparous groups, we found a 1.7-fold increased risk for overall mortality in the PPBC group (HR, 1.72 [95% CI, 1.17-2.52]; *P* = .006) (eTable 5 in Supplement 1). In multivariate analysis, this increased risk persisted after controlling for tumor stage, ER status, PV type, and age at diagnosis (HR, 1.56 [95% CI, 1.05-2.30]; *P* = .03) (Table 2). There was no association of increased mortality for the PPBC less than 5 years vs nulliparous groups in univariate analysis (HR, 1.37 [95% CI, 0.94-2.01]; *P* = .11) (eTable 5 in Supplement 1) or adjusted analysis (HR, 1.27 [95% CI, 0.86-1.86]; *P* = .23) (Table 2). No difference in mortality was observed between the parous BC at least 10 years post partum vs nulliparous groups by univariate analysis (HR, 1.23 [95% CI, 0.85-1.79]; *P* = .28) (eTable 5 in Supplement 1) nor adjusted analysis (HR, 1.09 [95% CI, 0.72-1.67]; *P* = .68) (Table 2).

**Table 2.** Multivariate Cox Proportional Hazard Regression Models for the Association of Breast Cancer Diagnosis and Time Since Most Recent Childbirth With All-Cause Mortality^a^

Group	All patients		ER-positive BC		ER-negative BC		*BRCA1* BC		*BRCA2* BC	
	HR (95% CI)	*P* value	HR (95% CI)	*P* value	HR (95% CI)	*P* value	HR (95% CI)	*P* value	HR (95% CI)	*P* value
**Time since most recent childbirth (3 categories)**											
Nulliparous (n = 224)	1 [Reference]	NA	1 [Reference]	NA	1 [Reference]	NA	1 [Reference]	NA	1 [Reference]	NA
PPBC 0 to <10 y (n = 419)	1.39 (0.98-1.97)	.06	2.28 (1.05-4.95)	.04	1.67(0.74-3.78)	.22	1.63 (0.98-2.74)	.06	1.20 (0.75-1.95)	.45
Parous ≥10 y (n = 260)	1.07 (0.70-1.63)	.75	1.23 (0.41-3.67)	.71	1.45 (0.53-3.93)	.47	1.14 (0.62-2.11)	.67	1.05 (0.58-1.88)	.88
**Time since most recent childbirth (4 categories)**											
Nulliparous (n = 224)	1 [Reference]	NA	1 [Reference]	NA	1 [Reference]	NA	1 [Reference]	NA	1 [Reference]	NA
PPBC 0 to <5 y (n = 228)	1.27 (0.86-1.86)	.23	2.35 (1.02-5.42)	.04	1.12 (0.44-2.83)	.81	1.39 (0.79-2.43)	.25	1.26 (0.73-2.16)	.41
PPBC 5 to <10 y (n = 191)	1.56 (1.05-2.30)	.03	2.18 (0.89-5.35)	.09	3.12 (1.22-7.97)	.02	2.03 (1.15-3.58)	.02	1.15 (0.66-2.00)	.63
Parous ≥10 y (n = 260)	1.09 (0.72-1.67)	.68	1.21 (0.40-3.65)	.73	1.80 (0.63-5.12)	.27	1.18 (0.64-2.18)	.60	1.03 (0.58-1.86)	.91

Abbreviations: HR, hazard ratio; NA, not applicable; PPBC, postpartum breast cancer.

^a^ HR results are adjusted for age (continuous variable) and stage (categorical variable).

### Associations Between Time Since Recent Childbirth and Survival by ER Status

**Figure 2.**
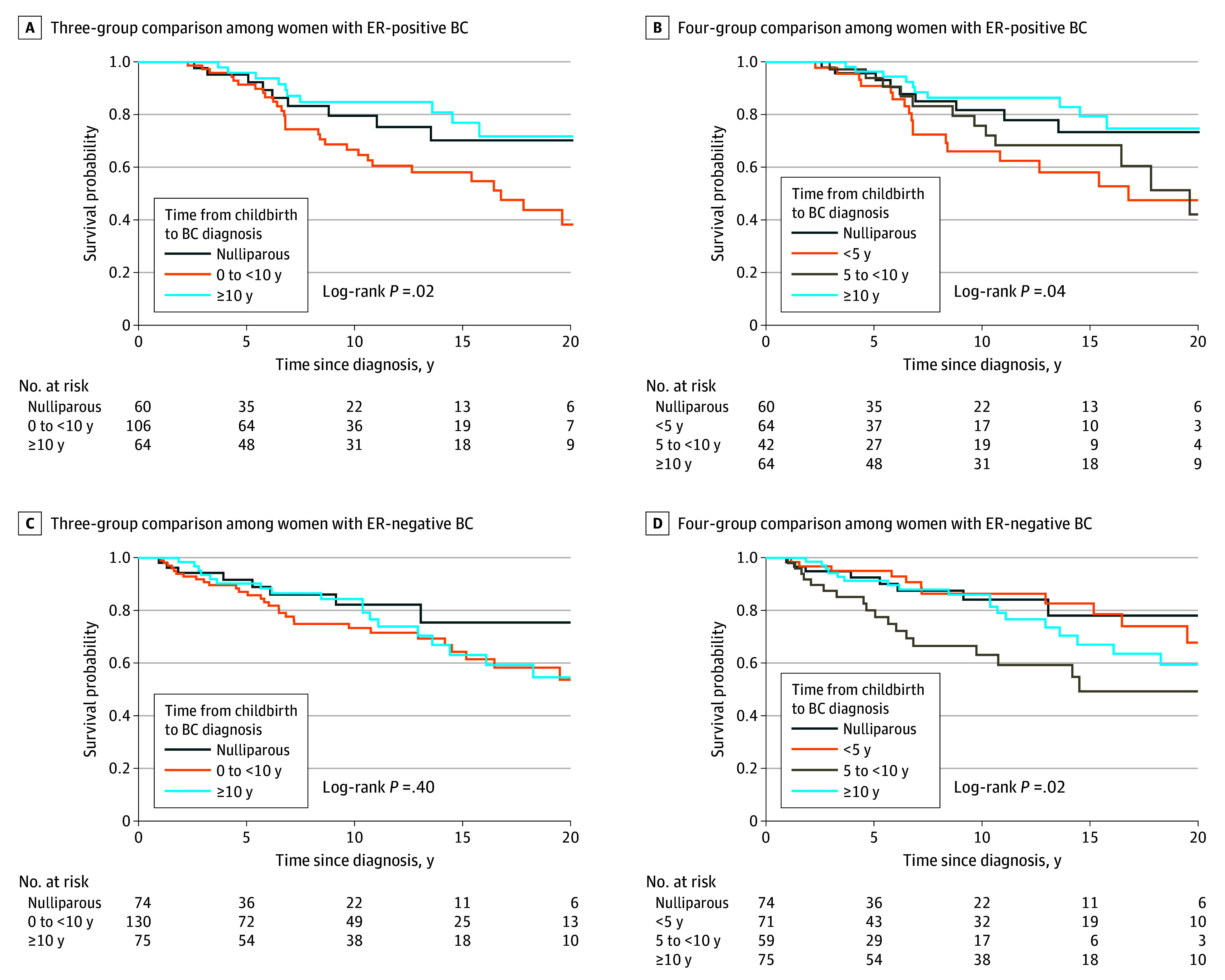
Survival Outcomes Among *BRCA1* and *BRCA2* Germline Pathogenic Variant Carriers With Breast Cancer (BC) by Estrogen Receptor (ER) Status and Time Since Recent Childbirth

In analysis restricted to women with ER-positive BC, a diagnosis of PPBC 0 to less than 10 years post partum was associated with more than 2-fold increased risk for overall mortality (HR, 2.28 [95% CI, 1.05-4.95]; *P* = .04) compared with nulliparous women (Figure 2A and Table 2). Further delineation of the postpartum cohort found that women diagnosed with ER-positive BC within less than 5 years of childbirth had the highest increased risk for overall mortality (HR, 2.35 [95% CI, 1.02-5.42]; *P* = .04) compared with nulliparous women (Figure 2B and Table 2). Women diagnosed with ER-positive BC at 5 to less than 10 years post partum did not have significantly increased risk for overall mortality compared with nulliparous women (HR: 2.18 [95% CI, 0.89-5.35]; *P* = .09) (Figure 2B and Table 2). Women diagnosed with ER-positive BC at least 10 years post partum had no statistically significant difference in overall mortality compared with nulliparous women (HR, 1.21 [95% CI, 0.40-3.65]; *P* = .73) (Figure 2B and Table 2).

In analysis restricted to women with ER-negative BC, poor prognosis was not evident when PPBC was assessed as 0 to less than 10 years (HR, 1.67 [95% CI, 0.74-3.78]; *P* = .22) (Figure 2C and Table 2). However, women with ER-negative BC diagnosed within 5 to less than 10 years of recent childbirth were 3 times more likely to die compared with nulliparous women (HR, 3.12 [95% CI, 1.22-7.97]; *P* = .02) (Figure 2D and Table 2). Consistent with the postpartum risk window being transient, and not an attribute of parity per se, and similar to that observed in ER-positive disease, women diagnosed at least 10 years post partum had no statistically significant difference for overall mortality compared with nulliparous women (HR, 1.80 [95% CI, 0.63-5.12]; *P* = .27) (Figure 2D and Table 2). Of note, we found 77.1% of *BRCA1* PV carriers were diagnosed with ER-negative tumors, and 75.6% of *BRCA2* carriers were diagnosed with ER-positive tumors. We next sought to determine whether the associations between time since recent childbirth and survival differed between *BRCA1* vs *BRCA2* carriers.

### Associations Between Time Since Recent Childbirth and Survival by *BRCA1* vs *BRCA2* Status

**Figure 3.**
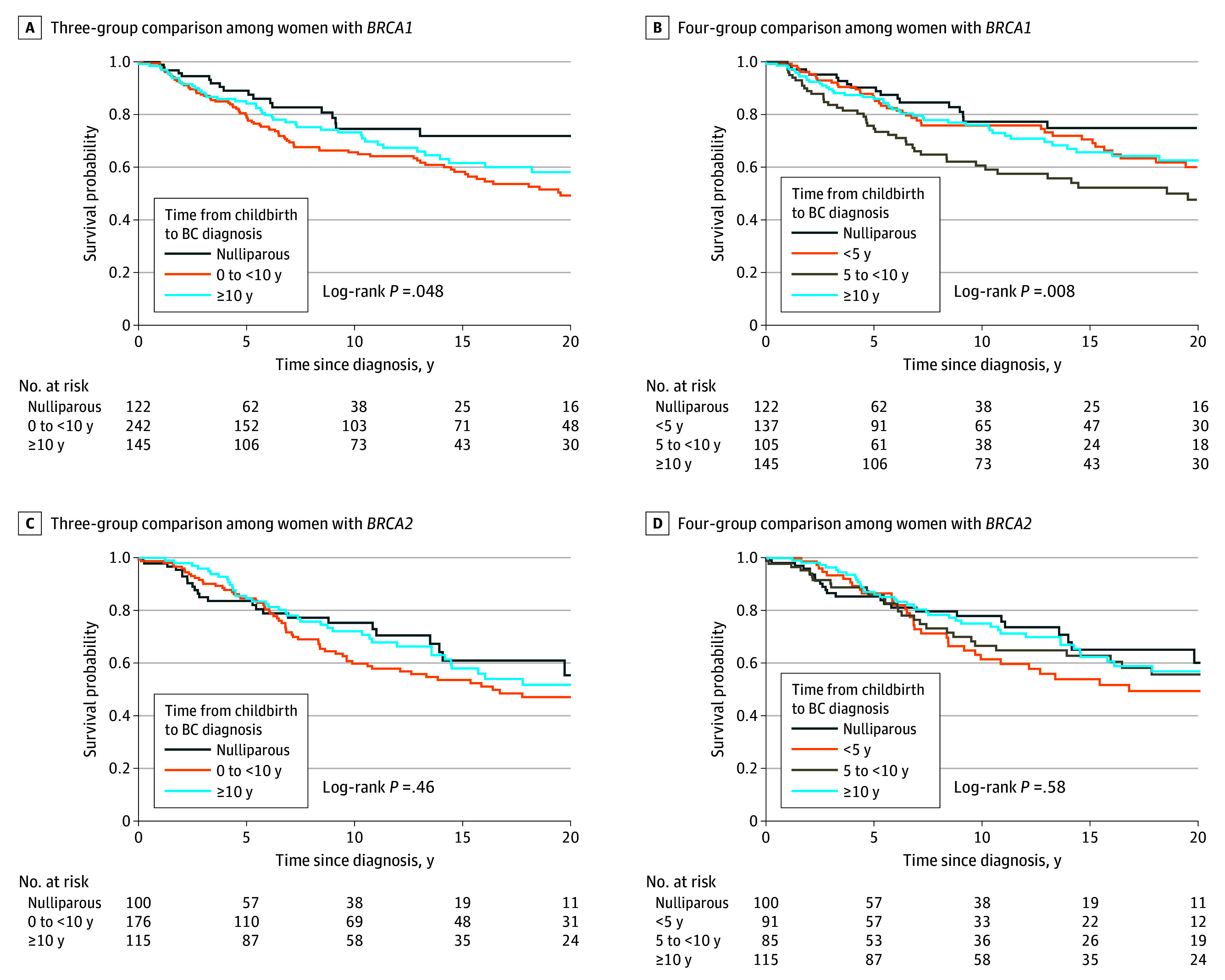
Survival Outcomes Among Women With Breast Cancer (BC) by *BRCA1* vs *BRCA2* Status and Time Since Recent Childbirth

In women with *BRCA1* diagnosed with PPBC at 0 to less than 10 years there was no association with overall poor prognosis compared with nulliparous women (HR, 1.63 [95% CI, 0.98-2.74]; *P* = .06) (Figure 3A and Table 2). When further delineating the postpartum cohort, a 2-fold increased risk in mortality was observed in the PPBC at 5 to less than 10 years group (HR, 2.03 [95% CI, 1.15-3.58]; *P* = .02) (Figure 3B and Table 2). The *BRCA1* parous 10 or more years group had no statistically significant difference for overall mortality compared with nulliparous women (HR, 1.18 [95% CI, 0.64-2.18]; *P* = .27) (Figure 3B and Table 2). In women with *BRCA2*, there were no associations with overall survival in women diagnosed in close proximity to recent childbirth in 3-group or 4-group or adjusted analyses (PPBC 0 to <10 years: HR, 1.20 [95% CI, 0.75-1.95]; *P* = .45; PPBC 0 to <5 years: HR, 1.26 [95% CI, 0.73-2.16]; PPBC 5 to <10 years: HR, 1.15 [95% CI, 0.66-2.00]; parous ≥10 years: HR, 1.03 [95% CI, 0.58-1.86]) compared with the nulliparous group (Figure 3 and Table 2).

To further investigate how proximity to recent childbirth may be a risk factor associated with poor prognosis in *BRCA1* carriers but not *BRCA2* carriers, we examined overall survival differences between women with *BRCA1* vs *BRCA2*. Survival between women with *BRCA1* vs *BRCA2* was not significantly different overall, nor when stratified by time since recent childbirth group (eTable 6 in Supplement 1). One potential explanation for the poor prognosis observed in *BRCA1* PVs is that *BRCA1* is differentially regulated across the pregnancy, lactation, and weaning cycle, such that its loss of function during this developmental window puts the gland at increased risk for disease progression. To begin to address this question, we used publicly available *Brca1/2* gene expression datasets obtained from murine models.^43,44,45^ We found peak levels of *Brca1* expression during the pregnancy cycle, whereas *Brca2* was not regulated across the reproductive cycle (eFigure 5 in Supplement 1).

### Associations Between Selected Reproductive Variables and Survival

We next evaluated whether reproductive risk factors other than time since recent childbirth were associated with BC survival in *BRCA1/2* carriers. Covariates included parity (0, 1, 2, and ≥3 births), age of first full-term birth (nulliparous, <21, 21-29, and ≥30 years), and age at last full-term birth (nulliparous, <21, 21-29, and ≥30 years). None were significantly associated with overall survival (eFigure 6 in Supplement 1). In sum, these analyses identify time since recent childbirth, but not the other reproductive factors, as a risk factor associated with reduced survival in *BRCA* carriers with BC.

## Discussion

In this cohort study, we present the association between reproductive risk factors and survival in young women with *BRCA1/2* BC with the goal of reducing mortality and increasing early detection of lethal *BRCA1/2*-driven BCs. Among germline *BRCA1/2* PV carriers, BC diagnosis within 5 to less than 10 years of childbirth was associated with elevated all-cause mortality overall in both ER-positive and ER-negative BCs. Furthermore, in this UK germline *BRCA1/2* BC cohort, the number of childbirths (parity), age at first full-term birth, and age at last full-term birth were not associated with increased mortality. However, we see that the overall birth trends have changed between 1950 and 2021, with fewer nulliparous women in earlier decades than later decades.

Women who carry a germline PV in the cancer predisposing genes *BRCA1 *or* BRCA2* have approximately 70% lifetime risk of developing BC.^37,46^ While numerous studies have examined reproductive risk factors that influence *BRCA1/2* BC rates with the goal of reducing incidence,^37,47,48^ here we assessed reproductive factors associated with survival. Our data are consistent with prior studies identifying a PPBC diagnosis as an independent risk factor associated with BC metastases, BC-specific death, and overall mortality across diverse BC populations.^22,23,26,27,28,29,30^ Furthermore, results of this study expand the understanding of PPBC by demonstrating increased risk for mortality in germline *BRCA* PV carriers diagnosed with PPBC. This study has implications for standard of care for women with *BRCA1/2* YOBC, as well as for genetic counseling of germline *BRCA1/2* PV carriers. Specifically, consideration for treatment escalation in ER-positive PPBC, and counseling for appropriate BC screening and risk reduction interventions in *BRCA1/2* carriers with recent childbirth may be warranted.

To date, the primary driver of BC metastasis in PPBC has been associated with tumor extrinsic factors, ie, the physiologically normal but tumor-supportive tissue microenvironment of the post partum involuting breast.^49^ These protumor stromal changes include the presence of activated fibroblasts,^50^ protumor collagen deposition,^50,51,52^ lymphangiogenesis,^53^ and immune infiltrate of immune suppressive and regulatory cells.^54,55,56,57,58^ Evidence that these physiologic stromal changes durably alter PPBC is supported by the distinct molecular and cellular profiles observed in PPBC compared with stage- and ER-subtype–matched tumors diagnosed in nulliparous women.^59,60^ Specifically, PPBC profiles are strongly associated with normal breast involution profiles, including increased tumor collagen fibrosis, lymphovascular invasion, immune infiltrate, and gene expression profiles characterized by immunosuppression and tumor cell invasion.^59,60^ Whether *BRCA* variant PPBC tumors display a distinct molecular profile consistent with involution and disease progression, as expected based on the poor outcomes observed in this UK cohort, remains to be determined.

We found the ER status of the tumors in this YOBC cohort was strongly delineated between *BRCA1* and *BRCA2* carriers, aligning well with previous reports of differential ER status between *BRCA1/2* carriers overall.^61,62^ In our study, 77.1% of *BRCA1* PV carriers were diagnosed with ER-negative tumors, and 75.6% of *BRCA2* carriers were diagnosed with ER-positive tumors. Of note, the ratio of ER-positive to ER-negative tumors in *BRCA2* carriers mirrors the general, nonfamilial BC population, ie, approximately 75% ER-positive and 25% ER-negative. One interpretation is that *BRCA2* interfaces with BC downstream of factors that determine ER subtype. Conversely, *BRCA1* carriers have more than 3.3-fold increased probability of having ER-negative disease compared with the general BC population, which suggests that loss of *BRCA1* function may be a contributing factor to the development of ER-negative BC. Indeed, *BRCA1*-associated BC is suggested to originate from luminal epithelial progenitors, a predominantly ER-negative cell population.^63,64^ In mice, loss of *Brca1* function inhibits the differentiation of ER-negative luminal progenitor cells into ER-positive epithelial cells,^65^ and promotes the expansion of ER-negative luminal progenitors in mammary tissue.^64,66,67^ Conditional knockout of *Brca1* function results in the development of mammary tumors with characteristics similar to *BRCA1* human tumors.^63^ These results suggest that loss of *BRCA1* function may result in accumulation of ER-negative luminal progenitor cells vulnerable for oncogenic transformation, and provide a rationale for the increased incidence of ER-negative disease observed in *BRCA1* carriers.

Of note, we did not see an increase in ER-negative disease in our postpartum *BRCA1* or *BRCA2* cohorts compared with the nulliparous patients. These data suggest that increased ER-negative disease does not account for the poor prognosis of PPBC in this cohort, a result similar to that reported in other PPBC studies.^22,23,28^ More research is needed to clarify potential relationships between mortality associated with BC diagnosis in close proximity to recent childbirth and BC subtypes overall and in *BRCA* carriers specifically.

Our study also found that ER-positive and ER-negative disease had different postpartum windows of risk. Because BC latency is thought to be greater than 5 years from initiation to overt cancer,^68,69^ the increase in poor prognostic ER-positive cases diagnosed within 5 years of child birth is consistent with promotion of preexisting subclinical tumors. The patients with ER-negative disease had poorest prognosis if diagnosed 5 to less than 10 years post partum, which could indicate promotion of existing as well as initiation of BC. Of note, there is evidence for increased *BRCA1* expression during pregnancy,^70,71^ consistent with a role in alveolar expansion.^66,67^ During the high-proliferative window of pregnancy, loss of *BRCA1* function might exacerbate DNA damage, given its critical role in DNA damage surveillance, and lead to increased oncogenic transformation. Furthermore, receptor activator of nuclear factor-κB (RANK) and its ligand (RANK-L) play an essential role in breast development during pregnancy^72,73^ and RANK and RANK-L have been shown to promote BC in mice with a *Brca1* variant.^74^ These preclinical studies are consistent with the idea that *BRCA1* may have unique functions during a reproductive cycle. *BRCA1* also regulates p53-dependent gene expression,^75^ and cooccurrence of somatic *TP53* PVs is more commonly observed with *BRCA1* PVs, compared with *BRCA2* PVs.^76^ Furthermore, mammary-specific deletion of Tp53 and *Brca1* leads to the development of murine mammary tumors having genomic and transcriptomic similarities to human basal-like BC.^77^ These potential biologic mechanisms linking *BRCA1* to lobule expansion during pregnancy may offer insights into why the peak risk window for poor prognosis in PPBC among *BRCA1 *PV carriers was observed later.

Our findings of no significant association between *BRCA2* and increased risk of mortality in PPBC may suggest that the overall increase in mortality observed in the combined *BRCA1/2* PPBC analysis was primarily associated with *BRCA1* carriers. However, for *BRCA2* carriers, our study may be underpowered to fully investigate the outcomes associated with *BRCA2* in PPBC, necessitating further research to address the association between *BRCA2* and PPBC mortality.

Our study also implicates a postpartum diagnosis, rather than the germline presence of *BRCA1/2* PVs, as the key factor associated with worse mortality in PPBC. Although *BRCA1* and *BRCA2* gene PVs represent more than 50% of all gene PVs associated with YOBC,^78^ it has been reported that patients with YOBC with *BRCA1/2* PVs have survival rates similar to patients with YOBC without these PVs.^79,80^ Furthermore, in this UK cohort, we found no significant difference in 20-year overall mortality between *BRCA1* vs *BRCA 2* PV carriers with YOBC; while finding a diagnosis at less than 10 years post partum was associated with higher risk of mortality compared with nulliparous women or in women diagnosed 10 or more years after childbirth. These results suggest that proximity to recent childbirth less than 10 years before BC diagnosis likely has a more pronounced impact on mortality in YOBC than the presence of a *BRCA* germline PV.

### Strengths and Limitations

Strengths of our study include the large sample size of patients with YOBC with germline *BRCA1/2* PVs and the availability of long-term follow-up data. Furthermore, we have rigorous data on the time between recent childbirth and BC diagnosis, a variable frequently missing from many BC databases, including The Cancer Genome Atlas Program and Metabric databases. Another study strength is that we have DNA sequencing data for the *BRCA1* and *BRCA2* genes, allowing us to identify the range and nature of PVs present. Furthermore, we have a sufficient number of combined *BRCA1 *and* BRCA2* PVs with confirmed ER status to perform analysis of the mortality risk stratified by time since recent childbirth group and ER subtype. However, since we are missing ER status on 44% of our cohort, selection bias cannot be ruled out. Another study limitation is that the data set was underpowered to conduct separate analyses for *BRCA1* vs *BRCA2* PVs carriers stratified by ER status. Furthermore, rigorous evaluation of *ERBB2* status by reproductive category was not possible due to the lack of *ERBB2* clinical data. We also do not have treatment data, which could impact overall mortality if treatment was different among groups. However, because this study comprises a geographically homogeneous population of patients with BC in the Northern England area, disparities in treatment approaches across groups may be minimized. Furthermore, the wide range of diagnosis eras and available treatment may potentially affect meaningful comparisons. Another study limitation is that we do not have race and ethnicity data or BC-specific mortality from the UK population, which may limit the generalization of the results.

## Conclusions

The findings of this cohort study suggest that germline *BRCA* PV carriers were at increased risk for all-cause mortality when BC was diagnosed within 5 to less than 10 years of recent childbirth, compared with nulliparous women and those diagnosed at least 10 years post partum. These findings are similar to the general BC population, where increased risk of metastasis and poor survival is observed in women diagnosed within 10 years of childbirth. For patients with *BRCA1*, the risk of increased mortality was especially significant when diagnosed at 5 to less than 10 years post partum. This delayed risk window may suggest an interaction between *BRCA1* and a pregnancy cycle that results in initiation of new cancers, in addition to the promotion of existing, subclinical tumors. Further research is needed to address this possibility. In sum, consideration of the potential impact of childbirth on BC outcomes in young germline *BRCA* PV carriers may improve standard of care within the realms of genetic counseling, disease prevention, and the clinic.

## Notes

Supplement 1. **eFigure 1.** Analytic Cohort Flowchart **eFigure 2.** Survival Comparison Between Those Who Had vs Had Not Oophorectomy and Mastectomy Before Breast Cancer Diagnosis **eFigure 3.** Survival Difference Between Nulliparous Patients Having First Childbirth After Breast Cancer Diagnosis vs the Rest of the Nulliparous Patients vs All Nulliparous Patients **eFigure 4. **Survival Difference by Year of Diagnosis Category Among Nulliparous Individuals **eFigure 5.** Evaluating *Brca1/2* Gene Expression Public Available Data From Murine Mammary Glands **eFigure 6.** Survival Outcome by Reproductive Variable Status and Time Since Recent Childbirth Groups **eTable 1.** Multivariate Cox Proportional Hazard Regression Models for All-Cause Mortality Among Women ≤45 Years Old With *BRCA* Pathogenic Variants **eTable 2.** Missing Data Percentage for Variables With Missing Values **eTable 3.** Comparing PPBC Status and Survival Among Individuals With Missing Data vs Nonmissing Data by Examined Variables **eTable 4.** Demographic and Clinical Characteristics of Analytic Cohort by *BRCA* Pathogenic Variation Status Group **eTable 5.** Unadjusted and Age-Adjusted Cox Proportional Hazard Regression Models for the Associations Between Breast Cancer Diagnosis Time Since First Childbirth Status and Survival **eTable 6.** Log-Rank Test Comparing *BRCA* Pathogenic Variants Status (*BRCA1* vs *BRCA2*) and All-Cause Mortality

Supplement 2.
Data Sharing Statement
