# Supplementary material for: Postpartum Breast Cancer and Survival in Women With Germline BRCA Pathogenic Variants
Source: JAMA Netw Open. 2024 Apr 19;7(4):e247421. doi: 10.1001/jamanetworkopen.2024.7421 (PMC11031688; doi:10.1001/jamanetworkopen.2024.7421)
Supplement: Supplement 1. — eFigure 1. Analytic Cohort Flowchart eFigure 2. Survival Comparison Between Those Who Had vs Had Not Oophorectomy and Mastectomy Before Breast Cancer Diagnosis eFigure 3. Survival Difference Between Nulliparous Patients Having First Childbirth After Breast Cancer Diagnosis vs the Rest of the Nulliparous Patients vs All Nulliparous Patients eFigure 4. Survival Difference by Year of Diagnosis Category Among Nulliparous Individuals eFigure 5. Evaluating Brca1/2 Gene Expression Public Available Data From Murine Mammary Glands eFigure 6. Survival Outcome by Reproductive Variable Status and Time Since Recent Childbirth Groups eTable 1. Multivariate Cox Proportional Hazard Regression Models for All-Cause Mortality Among Women ≤45 Years Old With BRCA Pathogenic Variants eTable 2. Missing Data Percentage for Variables With Missing Values eTable 3. Comparing PPBC Status and Survival Among Individuals With Missing Data vs Nonmissing Data by Examined Variables eTable 4. Demographic and Clinical Characteristics of Analytic Cohort by BRCA Pathogenic Variation Status Group eTable 5. Unadjusted and Age-Adjusted Cox Proportional Hazard Regression Models for the Associations Between Breast Cancer Diagnosis Time Since First Childbirth Status and Survival eTable 6. Log-Rank Test Comparing BRCA Pathogenic Variants Status (BRCA1 vs BRCA2) and All-Cause Mortality [file jamanetwopen-e247421-s001.pdf]

Supplemental Online Content

Zhang Z, Ye S, Bernhardt SM, et al. Postpartum breast cancer and survival in women with germline *BRCA* pathogenic variants. *JAMA Netw Open*. 2024;7(4):e247421. doi:10.1001/jamanetworkopen.2024.7421

eFigure 1. Analytic Cohort Flowchart

eFigure 2. Survival Comparison Between Those Who Had vs Had Not Oophorectomy and Mastectomy Before Breast Cancer Diagnosis

eFigure 3. Survival Difference Between Nulliparous Patients Having First Childbirth After Breast Cancer Diagnosis vs the Rest of the Nulliparous Patients vs All Nulliparous Patients

eFigure 4. Survival Difference by Year of Diagnosis Category Among Nulliparous Individuals

eFigure 5. Evaluating Brca1/2 Gene Expression Public Available Data From Murine Mammary Glands

eFigure 6. Survival Outcome by Reproductive Variable Status and Time Since Recent Childbirth Groups

eTable 1. Multivariate Cox Proportional Hazard Regression Models for All-Cause Mortality Among Women ⩽45 Years Old With BRCA Pathogenic Variants

eTable 2. Missing Data Percentage for Variables With Missing Values

eTable 3. Comparing PPBC Status and Survival Among Individuals With Missing Data vs Nonmissing Data by Examined Variables

eTable 4. Demographic and Clinical Characteristics of Analytic Cohort by BRCA Pathogenic Variation Status Group

eTable 5. Unadjusted and Age-Adjusted Cox Proportional Hazard Regression Models for the Associations Between Breast Cancer Diagnosis Time Since First Childbirth Status and Survival

eTable 6. Log-Rank Test Comparing BRCA Pathogenic Variation Status (BRCA1 vs BRCA2) and All-Cause Mortality

This supplemental material has been provided by the authors to give readers additional information about their work.

© 2024 Zhang Z et al. *JAMA Network Open.*

© 2024 Zhang Z et al. *JAMA Network Open.*

eFigure 1. Analytic cohort flowchart

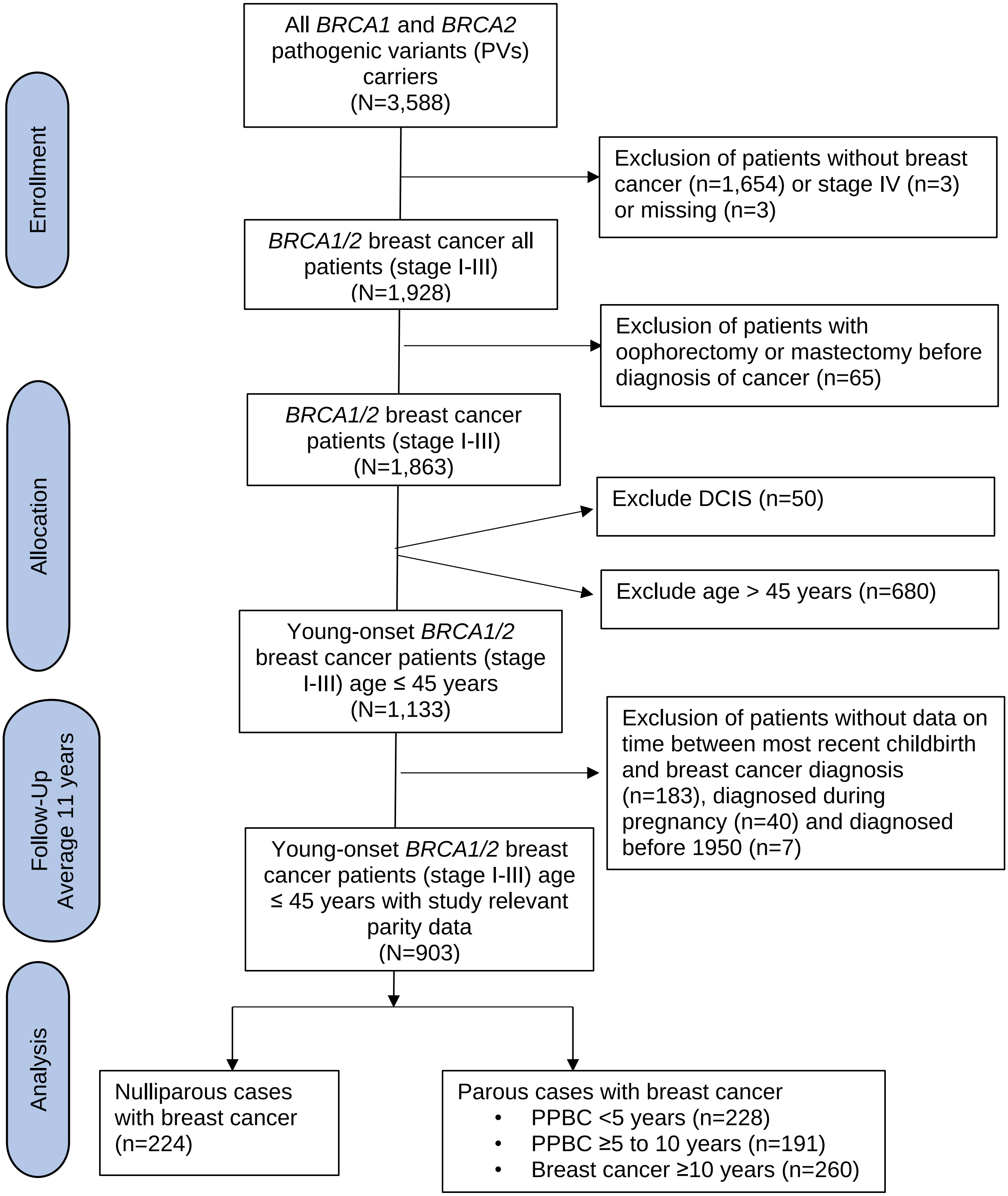

© 2024 Zhang Z et al. *JAMA Network Open.*

The flowchart illustrates the process to identify the final analytical cohort of N=903 eligible non-metastatic (stage I-III) breast cancer patients with germline *BRCA1* or *BRCA2* pathogenic variants (PVs). These patients had complete time-since-recent-childbirth data available during the follow-up period and were diagnosed at >15 and ≤ 45 years of age between 1950 and 2021.

© 2024 Zhang Z et al. *JAMA Network Open.*

eFigure 2: Survival comparison between those who had vs. had not oophorectomy and mastectomy before breast cancer diagnosis (p-value =0.30)

For the 65 patients who had oophorectomy and mastectomy before breast cancer, 6 nulliparous, 2 PPBC <5 years, 5 PPBC >=5 to 10 years, and 52 PPBC >=10.

Survival curve: 65 patients who had oophorectomy and mastectomy before breast cancer vs. the rest

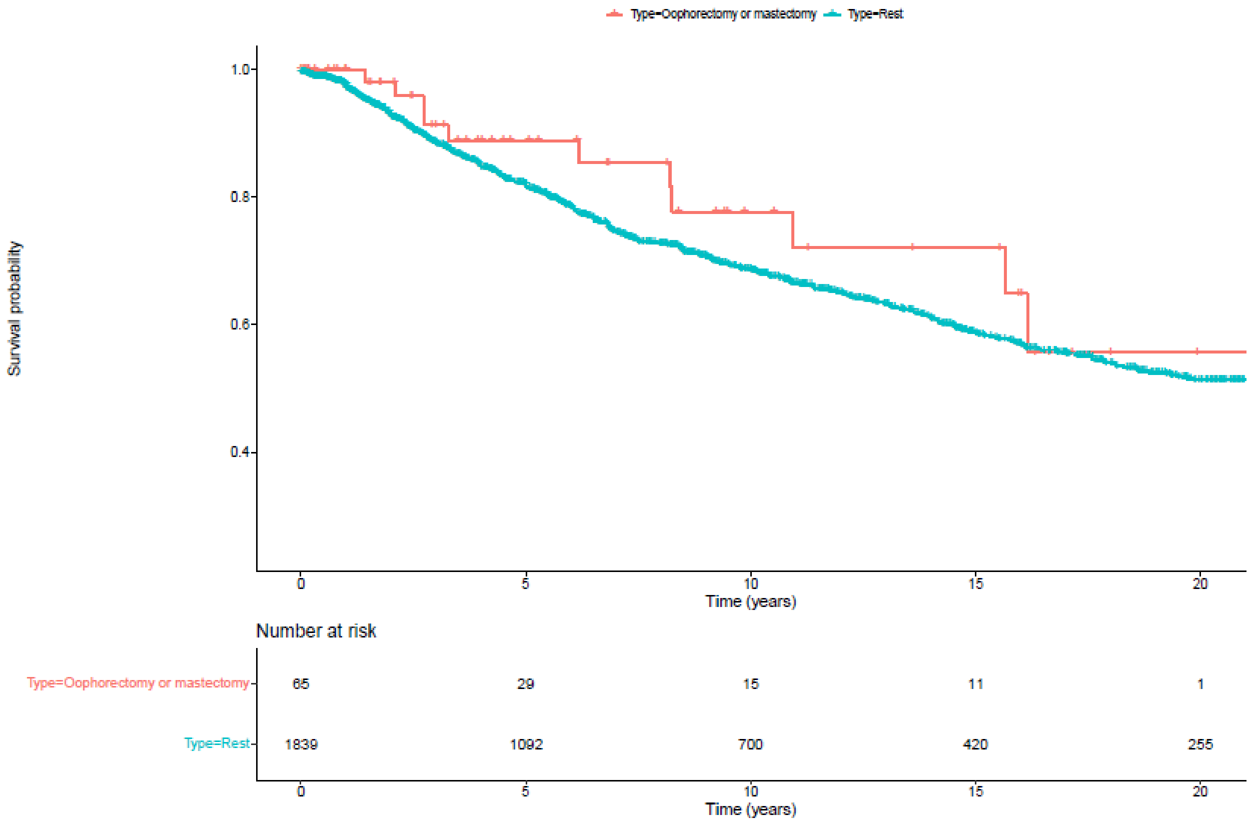

© 2024 Zhang Z et al. *JAMA Network Open.*

eFigure 3: Survival difference between nulliparous patients having the first childbirth after breast cancer diagnosis vs. the rest of the nulliparous patients vs. all nulliparous patients

Adjustment for Multiple Comparisons for the Logrank Test		
**Comparison Group**		**Tukey_Kramer P-value**
**1st childbirth after breast cancer diagnosis (n=10)**	**All nulliparous excluding those 10 cases (n=212)**	0.82
**1st childbirth after breast cancer diagnosis (n=10)**	**All nulliparous including those 10 cases (n=222)**	0.95
**All nulliparous excluding those 10 cases (n=212)**	**All nulliparous including those 10 cases (n=222)**	0.98

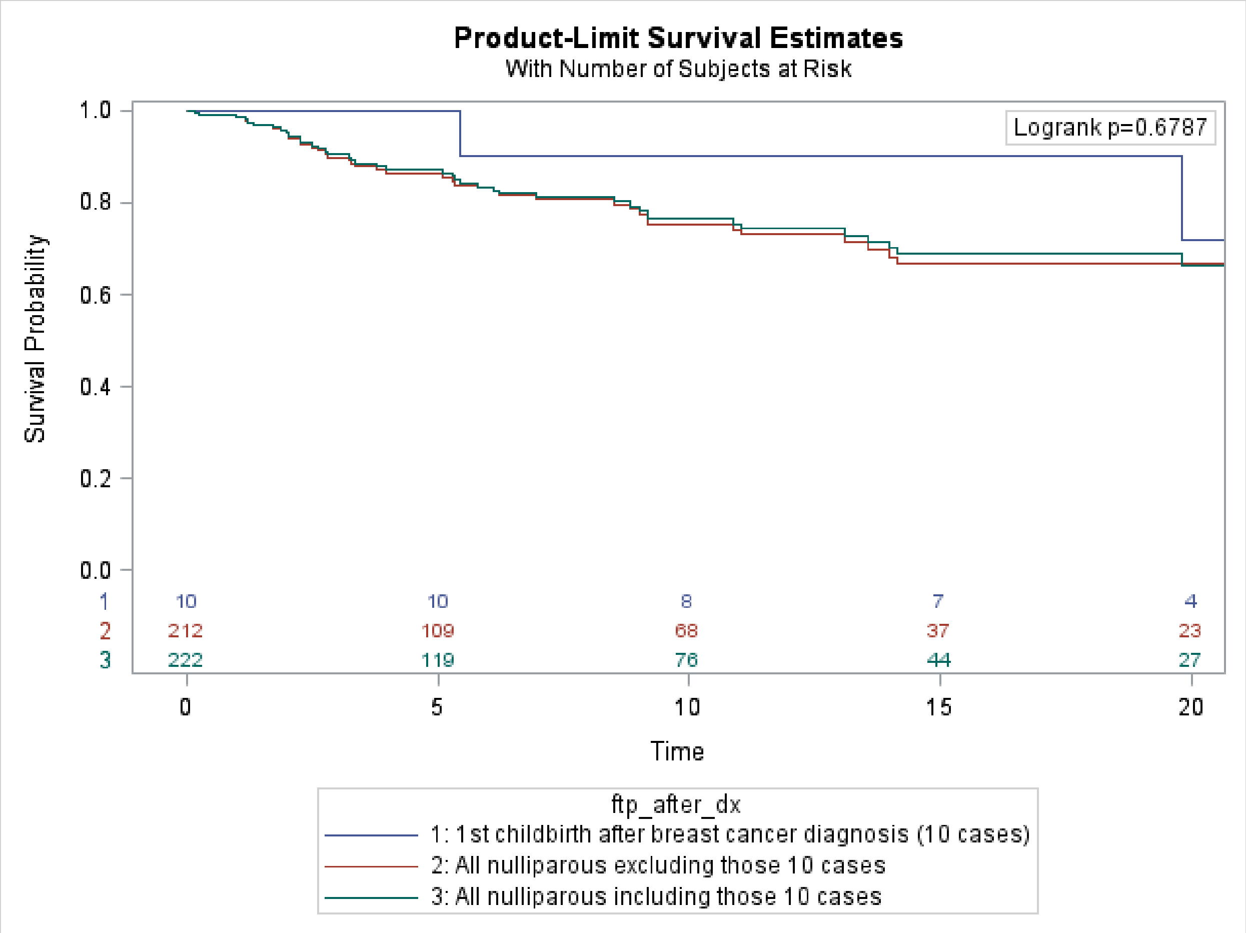

Reproductive Characteristics of the 10 Nulliparous Cases with Children after Diagnosis	
**Parity (Parous individuals only)**	**N (%)**
1	7 (70.0b)
2	3 (30.0)
≥ 3	0 (0)
**Age at First Full-term Birth (Parous individuals only)**	

© 2024 Zhang Z et al. *JAMA Network Open.*

<21	0 (0)
21-29	1 (10.0)
30-39	8 (80.0)
40+	1 (10.0)
**Age at Last Full-term Birth (Parous individuals only)**	
<21	0 (0.0)
21-29	1 (10.0)
30-39	7 (70.0)
40+	2 (20.0)

eFigure 4: Survival difference by year of diagnosis category among nulliparous individuals

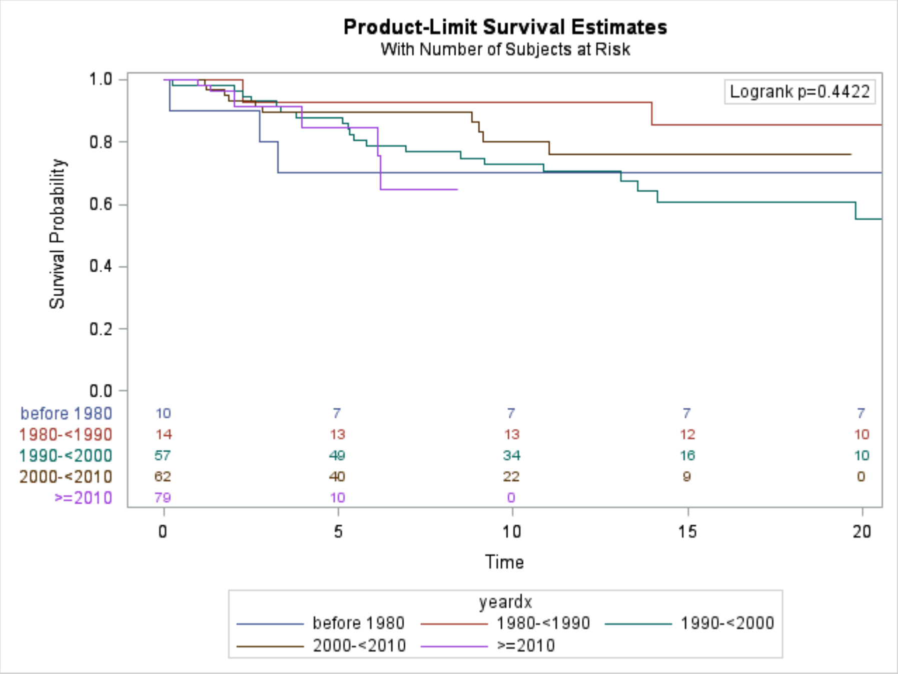

© 2024 Zhang Z et al. *JAMA Network Open.*

eFigure 5: Evaluating Brca1/2 gene expression public available data from murine mammary glands

Expression of BRCA genes using mouse VPLIR microarray datasets

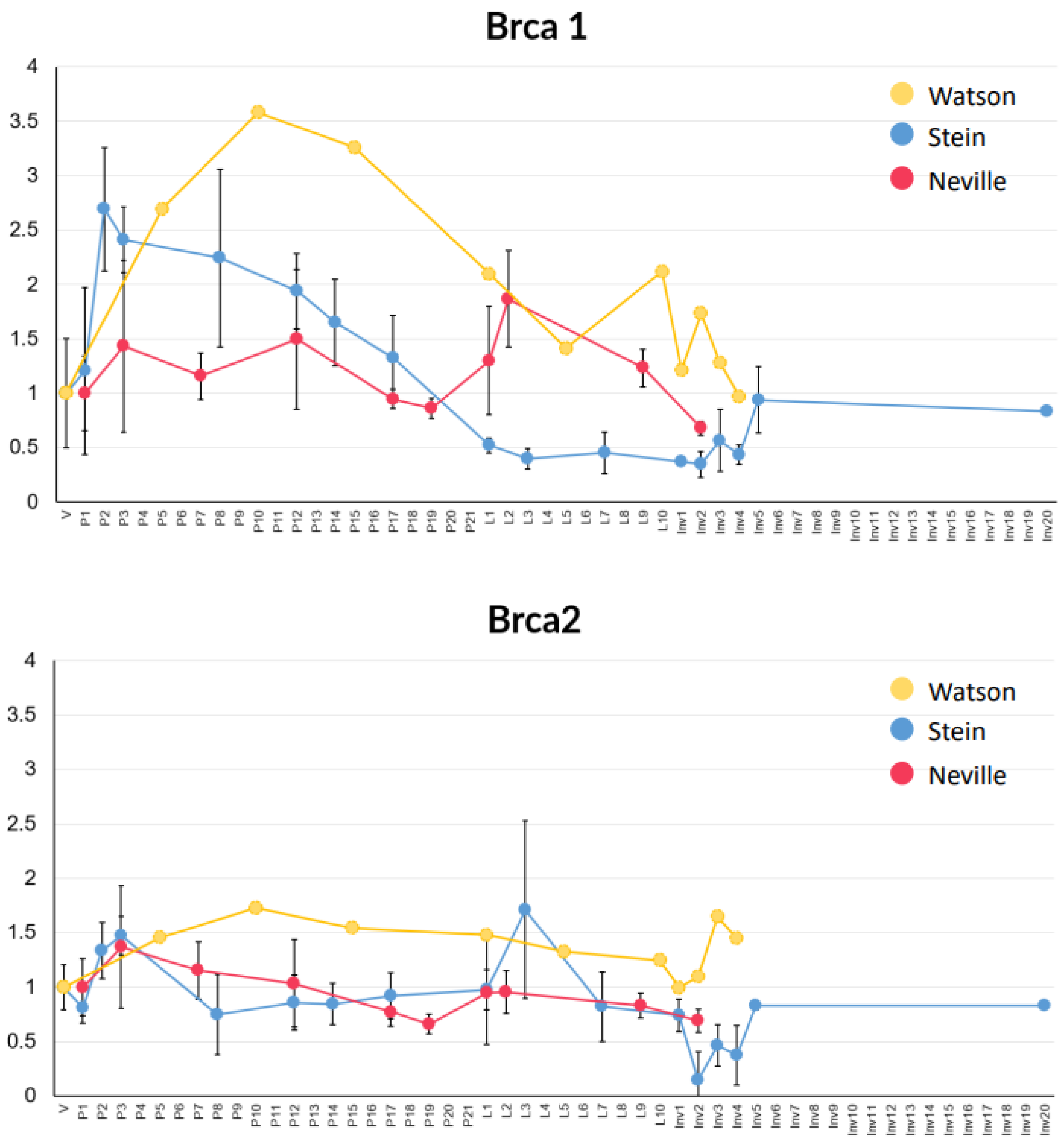

Gene expression data were obtained from previously published microarray data. Data obtained from Neville (2003) are normalized to gene expression at day 1 of pregnancy (P1); data obtained from Watson (2004) and Stein (2004) are normalized to gene expression in virgin mice

© 2024 Zhang Z et al. *JAMA Network Open.*

eFigure 6. Survival outcome by reproductive variable status and time-since-recent-childbirth groups

A.

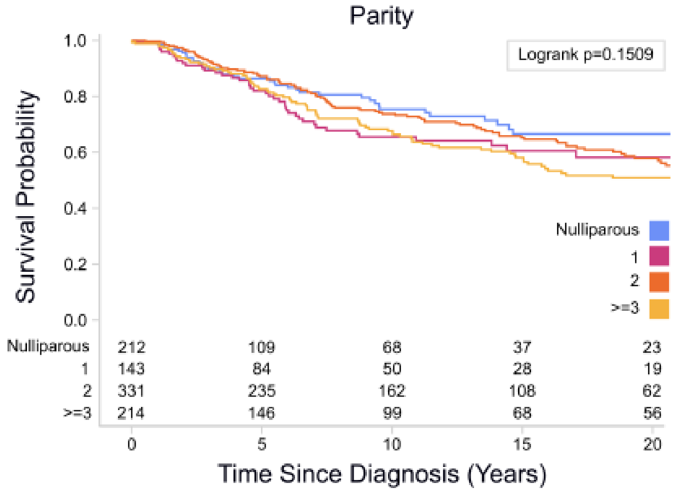

B.

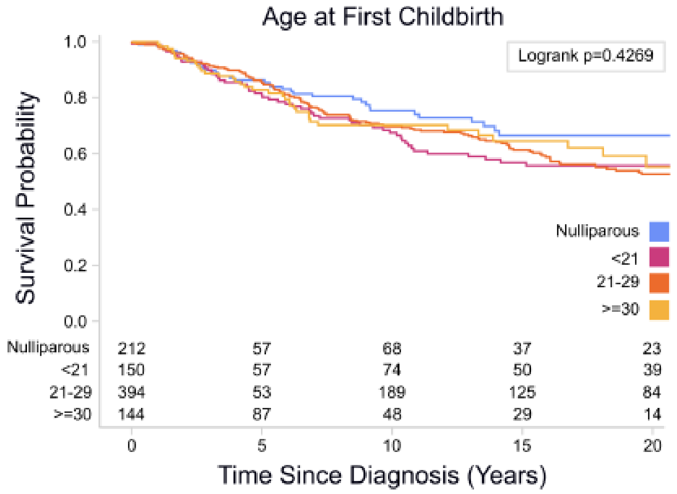

C.

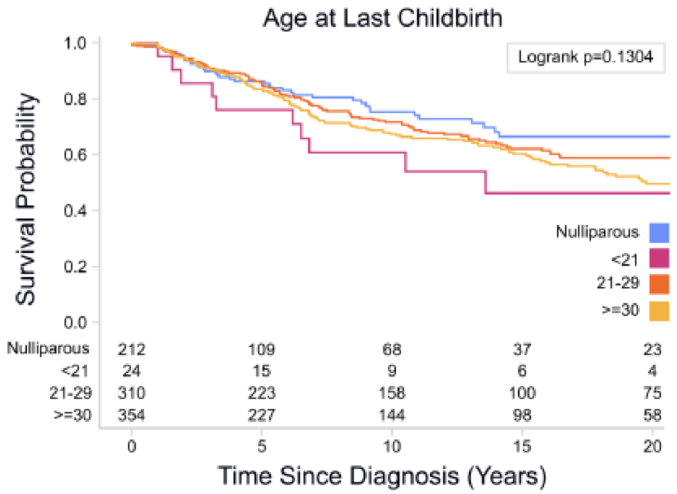

© 2024 Zhang Z et al. *JAMA Network Open.*

Survival outcomes by selected reproductive variables and time-since-recent-childbirth.

A. Parity: Different parity groups are represented by blue (nulliparous), pink (parity=1), dark orange (parity=2) and light orange (parity ≥3). **B. Age at first childbirth:** Different parity groups are represented by blue (nulliparous), pink (age at first childbirth<21), dark orange (age at first childbirth 21-29) and light orange (age at first childbirth≥30). **C. Age at last childbirth:** Different parity groups are represented by blue (nulliparous), pink (age at last childbirth <21 years old), dark orange (age at last childbirth 21-29 years old) and light orange (age at last childbirth ≥30 years old)

© 2024 Zhang Z et al. *JAMA Network Open.*

eTable 1. Multivariate Cox proportional hazard regression (HR) models for all-cause mortality among women ≤45 years old with BRCA pathogenic variants

**Participant Characteristics**	HR (95% CI)
**Time since most recent childbirth**	
Nulliparous	1.00 (ref)
PPBC 0-<5 years	1.24 (0.84-1.84)
PPBC 5-<10 years	1.52 (1.03-2.26)
Parous ≥ 10 years	1.13 (0.74-1.72)
**Stage**	
1	1.00 (ref)
2	1.38 (0.85-2.25)
3	5.89 (2.73-12.72)
**Age at breast cancer diagnosis**	1.01 (0.98-1.04)
**Year at diagnosis**	
Before 1980	1.08 (0.60-1.94)
1980-1990	0.68 (0.37-1.23)
1990-2000	0.92 (0.54-1.55)
2000-2010	0.60 (0.34-1.06)
After 2010	1.00 (ref)

© 2024 Zhang Z et al. *JAMA Network Open.*

eTable 2. Missing Data Percentage for Variables with Missing Values

	No. (%)
**Estrogen status**	
ER+	231 (25.6)
ER-	279 (30.9)
Missing	393 (43.5)
**Tumor size**	
0.1—≤2.0 cm	193 (21.4)
>2.0—≤5.0 cm	146 (16.2)
>5.0 cm	4 (0.4)
Missing	560 (62.0)
**Histology Grade**	
I	6 (0.7)
II	103 (11.4)
III	460 (50.9)
Missing	334 (37.0)
**Stage**	
1	150 (16.6)
2	186 (20.6)
3	17 (1.9)
Missing	550 (60.9)
**Age at menarche**	
≤13	248 (27.5)
>13	135 (15.0)
Missing	131 (57.6)

© 2024 Zhang Z et al. *JAMA Network Open.*

eTable 3a. Comparing PPBC status among individuals with missing data vs. non-missing data by examined variables

	Nulliparous (*N*=224, 24.8%) [a]	PPBC <5 (*N*=228, 25.2%)	PPBC 5-<10 (*N*=191, 21.2%)	PPBC ≥10 (*N*=260, 28.8%)	P value
	**No. (%)**	**No. (%)**	**No. (%)**	**No. (%)**	
**Estrogen status**					0.26
Not Missing	135 (60.3)	135 (59.2)	101 (52.9)	139 (53.5)	
Missing	89 (39.7)	93 (40.8)	90 (47.1)	121 (46.5)	
**Tumor size**					0.61
Not Missing	92 (41.1)	89 (39.0)	68 (35.6)	94 (36.1))	
Missing	132 (58.9)	139 (61.0)	123 (64.4)	166 (63.9)	
**Histology Grade**					0.22
Not Missing	149 (66.5)	151 (66.2)	114 (59.7)	155 (59.6)	
Missing	75 (33.5)	77 (33.8)	77 (40.3)	105 (40.4)	
**Stage**					0.91
Not Missing	91 (40.6)	90 (39.5)	71 (37.2)	101 (38.8)	
Missing	133 (59.4)	138 (60.5)	120 (62.8)	159 (61.2)	
**Age at menarche**					0.46
Not Missing	91 (40.6)	97 (42.5)	75 (39.3)	120 (46.2)	
Missing	133 (59.4)	131 (57.5)	116 (60.7)	140 (53.8)	

eTable 3b. Comparing survival among individuals with missing data vs. non-missing data by examined variables

	Alive	Death	P value
	**No. (%)**	**No. (%)**	
**Estrogen status**			<0.001
Not Missing	393 (67.2)	117 (36.8)	
Missing	192 (32.8)	201 (63.2)	
**Tumor size**			<0.001
Not Missing	261 (44.6)	82 (25.8)	
Missing	324 (55.4)	236 (74.2)	
**Histology Grade**			<0.001
Not Missing	419 (71.6)	150 (47.2)	
Missing	166 (28.4)	168 (52.8)	
**Stage**			<0.001
Not Missing	267 (45.6)	86 (27.0)	
Missing	318 (59.4)	232 (73.0)	

© 2024 Zhang Z et al. *JAMA Network Open.*

**Age at menarche**			<0.001
Not Missing	291 (49.7)	92 (28.9)	
Missing	294 (50.3)	226 (71.1)	

© 2024 Zhang Z et al. *JAMA Network Open.*

eTable 4. Demographic and Clinical Characteristics of Analytic Cohort by *BRCA* Pathogenic Variants Status Group

	***BRCA1* (*N*=509, 56%)** [(a)]	***BRCA2* (*N*=394, 44%)**	**P value**
	**No. (%)**	**No. (%)**	
**Mean age at diagnosis (SD)**	36.9 (5.5)	37.8 (5.2)	0.06[(b)]
**Estrogen status**			<0.001 [(c)(d)]
ER+	67 (22.9)	164 (75.6)	
ER-	226 (77.1)	53 (24.4)	
Missing	216	177	
**Tumor size**			0.84[(c)(d)]
0.1—≤2.0 cm	102 (54.8)	91 (58.0)	
>2.0—≤5.0 cm	82 (44.1)	64 (40.8)	
>5.0 cm	2 (1.1)	2 (1.3)	
Missing	323	237	
**Histology Grade**			<0.0001[(c)(d)]
I	1 (0.3)	5 (2.1)	
II	18 (5.5)	85 (35.3)	
III	309 (94.2)	151 (62.7)	
Missing	181	153	
**Stage**			0.51[(c)(d)]
1	87 (45.1)	63 (39.4)	
2	98 (50.8)	88 (55.0)	
3	8 (4.2)	9 (5.6)	
Missing	316	234	
**Year of Diagnosis**			0.77[(c)]
Before 1980	65 (12.8)	42 (10.7)	
1980-1990	66 (13.0)	54 (13.7)	
1990-2000	132 (25.9)	114 (28.9)	
2000-2010	125 (24.6)	95 (24.1)	
After 2010	121 (23.8)	89 (22.6)	
**Parity**			0.04[(c)])
0	115 (22.6)	99 (25.1)	
1	93 (18.3)	51 (12.9)	
2	172 (33.8)	159 (40.4)	
≥ 3	129 (25.3)	85 (21.6)	

© 2024 Zhang Z et al. *JAMA Network Open.*

**Age at first FTP**			0.0003[c] (excluding nulliparous group)
<21	93 (23.6)	58 (19.7)	
21-29	224 (56.9)	170 (57.6)	
30-39	74 (18.8)	67 (22.7)	
40+	3 (0.8)	0 (0.0)	
**Age at last FTP**			0.61 [c] (excluding nulliparous group)
<21	15 (3.8)	10 (3.4)	
21-29	180 (45.7)	130 (44.1)	
30-39	187 (47.5)	150 (50.9)	
40+	12 (3.1)	5 (1.7)	
**Age at menarche**			0.14[c][d]
≤13	137 (61.7)	111 (68.9)	
>13	85 (38.3)	50 (31.1)	
Missing	287	233	
**Type of mutation**			<0.001[c]
Copy Number Variants (Large deletion + Large rearrangement)	92 (18.1)	20 (5.1)	
Truncating	360 (70.7)	345 (87.5)	
Splice site	34 (6.7)	18 (4.6)	
Missense	17 (3.3)	11 (2.8)	
Promotor	6 (1.2)	0 (0.0)	

Note:
[a] Patients gave birth after diagnosis (n=10) were included in the nulliparous group.
[b] Kruskal Wallis test
[c] Chi-Square test or Fisher's Exact test
[d] Missing value categories were excluded from P-value calculation.

© 2024 Zhang Z et al. *JAMA Network Open.*

eTable 5. Unadjusted and age-adjusted Cox proportional hazard regression (HR) models for the associations between breast cancer diagnosis time since first childbirth status and survival

	**Unadjusted**			**Age-adjusted**		
Time since First childbirth	HR (95% CI)	P	Overall P	HR (95% CI)	P	Overall P
**All Stages**						
Nulliparous	1.00 (Reference)	N/A	**0.048**	1.00 (Reference)	N/A	**0.037**
PPBC 0-<5 years	1.37 (0.94-2.01)	0.11		1.36 (0.93-1.99)	0.12	
Parous 5-<10 years	1.72 (1.17-2.52)	**0.006**		1.64 (1.11-2.42)	**0.01**	
Parous ≥ 10 years	1.23 (0.85-1.79)	0.28		1.09 (0.72-1.66)	0.69	
**Stages I & II**						
Nulliparous	1.00 (Reference)	N/A	**0.025**	1.00 (Reference)	N/A	**0.025**
PPBC 0-<5 years	1.09 (0.54-2.18)	0.81		1.11 (0.55-2.24)	0.76	
Parous 5-<10 years	1.90 (0.98-3.68)	0.058		2.04 (1.02-4.04)	**0.04**	
Parous ≥ 10 years	0.70 (0.35-1.40)	0.31		0.83 (0.37-1.90)	0.66	
**BRCA1**						
Nulliparous	1.00 (Reference)	N/A	**0.011**	1.00 (Reference)	N/A	**0.01**
PPBC 0-<5 years	1.48 (0.84-2.58)	0.17		1.42 (0.81-2.49)	0.17	
Parous 5-<10 years	2.41 (1.39-4.19)	**0.002**		2.22 (1.26-3.89)	**0.006**	
Parous ≥ 10 years	1.48 (0.85-2.56)	0.17		1.20 (0.65-2.22)	0.57	
**BRCA2**						
Nulliparous	1.00 (Reference)	N/A	0.613	1.00 (Reference)	N/A	0.59
PPBC 0-<5 years	1.38 (0.82-2.35)	0.23		1.39 (0.82-2.35)	0.23	
Parous 5-<10 years	1.19 (0.69-2.05)	0.54		1.18 (0.68-2.05)	0.56	
Parous ≥ 10 years	1.04 (0.63-1.74)	0.87		1.02 (0.57-1.83)	0.94	
**ER+**						
Nulliparous	1.00 (Reference)	N/A	**0.046**	1.00 (Reference)	N/A	**0.046**
PPBC 0-<5 years	2.15 (0.96-4.84)	0.06		2.19 (0.97-4.92)	0.06	
Parous 5-<10 years	1.77 (0.74-4.19)	0.20		1.90 (0.78-4.62)	0.16	
Parous ≥ 10years	0.81 (0.33-1.99)	0.64		1.00 (0.35-2.90)	1.00	
**ER-**						
Nulliparous	1.00 (Reference)	N/A	**0.027**	1.00 (Reference)	N/A	**0.027**
PPBC 0-<5 years	1.03 (0.42-2.57)	0.95		1.07 (0.43-2.70)	0.88	
Parous 5-<10 years	2.83 (1.23-6.52)	**0.014**		3.13 (1.26-7.74)	**0.014**	
Parous ≥ 10 years	1.48 (0.64-3.40)	0.36		1.74 (0.63-4.77)	0.28	

© 2024 Zhang Z et al. *JAMA Network Open.*

eTable 6: Log-Rank Test Comparing BRCA status (BRCA1 vs. BRCA2) and all-cause mortality

	P
All	0.48
Nulliparous	0.21
PPBC 0-<5 years	0.14
PPBC 5-<10 years	0.22
Parous ≥ 10 years	0.76

© 2024 Zhang Z et al. *JAMA Network Open.*
