# Supplementary material for: Postpartum Breast Cancer and Survival in Women With Germline BRCA Pathogenic Variants
Source: JAMA Netw Open. 2024 Apr 19;7(4):e247421. doi: 10.1001/jamanetworkopen.2024.7421 (PMC11031688; doi:10.1001/jamanetworkopen.2024.7421)
Supplement: Supplement 2. — Data Sharing Statement [file jamanetwopen-e247421-s002.pdf]

Data Sharing Statement

Zhang. Postpartum Breast Cancer and Survival in Women With Germline BRCA Pathogenic Variants. *JAMA Netw Open*. Published April 19, 2024.
doi:10.1001/jamanetworkopen.2024.7421

Data
Data available: Yes
Data types: Deidentified participant data
How to access data: zhanzh@ohsu.edu
When available: With publication

Supporting Documents
Document types: None

Additional Information
Who can access the data: Researchers whose proposed use of the data has been approved
Types of analyses: Epidemiology
Mechanisms of data availability: IRB approved research
